# Individual marking of soft-bodied subtidal invertebrates *in situ* – A novel staining technique applied to the giant plumose anemone *Metridium farcimen* (Tilesius, 1809)

**DOI:** 10.1371/journal.pone.0188263

**Published:** 2017-11-21

**Authors:** Christopher D. Wells, Kenneth P. Sebens

**Affiliations:** 1 Biology Department, University of Washington, Seattle, WA, United States of America; 2 Friday Harbor Laboratories, University of Washington, Friday Harbor, WA, United States of America; 3 School of Aquatic and Fishery Sciences, University of Washington, Seattle, WA, United States of America; University of California Irvine, UNITED STATES

## Abstract

The ability to recognize individuals and track growth over time is crucial to population dynamics research as well as studies of animal behavior. Invertebrates are particularly difficult to track as they often molt, have regenerative capabilities, or lack hard parts to attach markers. We tested, in laboratory and field studies, a new way of marking sea anemones (order Actiniaria) by injection of three vital stains (i.e., neutral red, methylene blue, and fluorescein). Neutral red and methylene blue did not affect growth or survival, but fluorescein was lethal at high concentrations. Marked individuals could be identified up to seven months after injection with neutral red, six weeks with methylene blue, and three days with low concentrations of fluorescein. Neutral red could be used for long-term monitoring of growth and survival in the field, and in combination with methylene blue could be used to mark individuals in distinguishable patterns for short-term studies such as examining predator-prey interactions, movement of individuals, and recruitment survival.

## Introduction

The ability to recognize individuals and track growth over time is crucial to population dynamics research, animal behavior studies, as well as to parameterize bioenergetics models [1, 2]. Several methods for marking individuals have been used on marine invertebrates including the use of inserted tags, external tags or colors applied to hard parts, and staining techniques (e.g., [3–6]). However, invertebrates often lack hard parts to attach markers, molt, or have regenerative capabilities and these methods frequently involve removing the animal from the field to mark them.

Only external staining techniques have been used to mark sea anemones (order Actiniaria) [3]. The method used by Sebens [3, 7–10] requires the anemone to either be exposed during a low tide or taken out into the air to apply the stain. For subtidal anemone species this would involve undue stress during the removal process and then the difficult task of reattachment in the same location. Additionally, the impact of the staining process and the impact of the stain on growth and survival of the anemones has never been quantified.

The objective of this research was to develop a technique to mark subtidal anemones *in situ* while minimizing short- and long-term effects on growth and survival. This method was developed for use in studies of the population dynamics and a bioenergetics model for the giant plumose anemone *Metridium farcimen* (Tilesius, 1809). In both the laboratory and field, we experimentally tested a novel method of marking sea anemones through injection of three vital stains (neutral red, methylene blue, and fluorescein).

## Materials and methods

### Laboratory experiment

Sixty three individuals of *M*. *farcimen* (2.2–6.6 cm diameter, 3.7 cm mean) were collected off the pontoons of the Port of Friday Harbor marina, Friday Harbor, Washington (48.538°N, 123.015°W) and maintained in three sea tables (98x98x12 cm) at Friday Harbor Laboratories. Collections were limited to 63 individuals to limit impact on the wild population and so the sea anemones were not overcrowded in the laboratory tank. Collections were authorized by the director of Friday Harbor Laboratories and by the port commissioners at the Port of Friday Harbor marina. This research did not involve any endangered or protected species. Anemones were maintained for two weeks before experimental treatments were applied to allow time for any pedal disk damage incurred during collection to heal. Seawater exchange was kept at 1.0 L/min during the healing period. This rate created a slow circular current, but was not fast enough to dislodge attached and attaching anemones. Anemones were fed 24-hr old *Artemia salina* (Linnaeus, 1758) nauplii daily.

One of three treatments (21 individuals per treatment) was randomly applied to each anemone through hypodermic injection with a 22-gauge, stainless steel needle. Anemones were injected with 1.0 mL of either 10% neutral red, 10% methylene blue, or with raw seawater alone as a control. All stains in this and the subsequent experiment were diluted in raw seawater. Both neutral red and methylene blue do not fully dissolve at this concentration in raw seawater and therefore some stain was injected in solid form. No work has been done to look at the effect of undissolved stain on animal tissues, although presumably solid stain would dissolve in the sea water in the coelenteron of the anemones and cause little damage. Anemones were injected about 0.5 cm above the pedal disk, directly into the coelenteron (i.e., gastric cavity). After anemones were injected, water exchange was increased to 1.5 L/min. A circulation pump (18.6 L/min) was added to each tank to increase the circular current on a six hour on, two hours off cycle.

Both neutral red and methylene blue are vital stains. Neutral red binds to lysosomes in live tissue, whereas methylene blue binds to DNA. Additionally methylene blue is used as an antimicrobial at very low concentrations and in a variety of fields of medicine [11]. Neutral red has been successfully applied to the outside of intertidal anemones [3], but its impact on growth and survival has yet to be quantified. Also, its efficacy to mark sea anemones when injected has not been tested.

Growth of anemones was monitored on a weekly basis for six weeks starting in July 2015 by measuring the major (i.e., maximum) and minor (i.e., perpendicular to the maximum) pedal disk diameters with digital calipers and calculating an average pedal disk diameter as described in Wells [12]. The effect of injecting neutral red and methylene blue on growth of *M*. *farcimen* was computed using a residual maximum likelihood linear mixed models in JMP 13 with days passed, tank, and individual anemone as random effects and solution injected as a fixed effect. Survival was checked daily.

### Field experiment

The previous experiment was repeated with small modifications to determine if the same growth and survival patterns seen in the lab would be observed in the field. On the underside of the 10 pontoons of the floating docks at Friday Harbor Laboratories, 50 specimens of *M*. *farcimen* (1.4–8.7 cm diameter, 3.1 cm average) were selected for one of five treatments (10 individuals per treatment with one individual on each pontoon). Number of anemones injected and subsequently measured was limited by dive-partner availability. Anemones were injected with 10% neutral red, 10% methylene blue, 10% fluorescein, or 0.25% fluorescein or raw seawater alone as a control during a SCUBA dive. Fluorescein is commonly used to observe water flow and fluoresces yellow-green. Anemones were injected with 2–6 mL of solution with larger anemones receiving more material. 16-gauge stainless steel needles were used to inject each anemone. Needle size was increased to reduce the chance of blockages in the needle from aggregates of undissolved stain, which can easily be corrected in a laboratory setting, but cannot while SCUBA diving. Anemones were otherwise not disturbed. Neighboring non-experimental anemones were not removed; density of potential competitors was not controlled.

Photographs of anemones were taken (GoPro Hero4 Black Edition) weekly for the first six weeks and then every two to three weeks thereafter starting in July 2015. From these photographs, major and minor pedal disk diameters could be measured in the program ImageJ (National Institute of Health), which allowed growth rates to be calculated as described earlier. As anemones did not move extensively during the experiment, control anemones (i.e., seawater-injected anemones) could be tracked without external markings based on their position compared to marked anemones and their size. Effect of solution injected on growth rate was computed as in the laboratory experiment except with the addition of pontoon as a random effect. Survival was checked one day after the initial treatment and then every time as photographs were taken. Anemones were considered dead if there was major tissue necrosis typical of anemone death.

## Results

**Fig 1:**
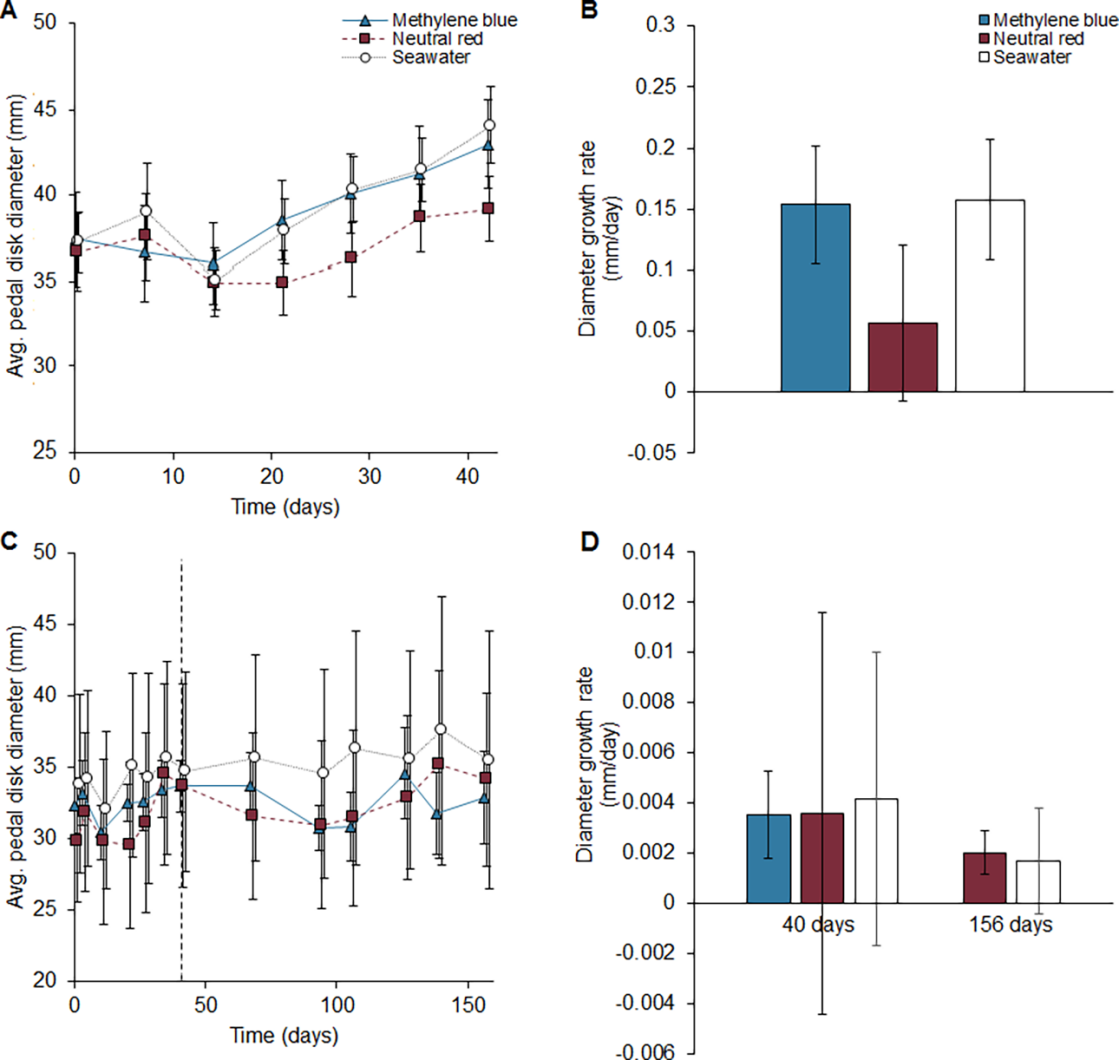
**Growth of *Metridium farcimen* injected with 10% neutral red, 10% methylene blue, or seawater (control) in (A, B) laboratory and (C, D) field experiments.** There was no significant difference between the marked anemone and control growth rates in either both laboratory or field experiments (p>0.05, n = 21 and n = 10 respectively). Values are means ± standard error. The dashed line in panel C indicates the time at which methylene blue marked individuals lost sufficient color to be identified as marked. Neutral red individuals were still clearly visible at 156 days. Growth rates were compared at 40 and 156 days between all treatments that had visibly marked individuals.

Neither methylene blue nor neutral red had significant effects on growth in both laboratory and field experiments (p>0.05 for both, Fig 1), although sample size was small in the field experiment (n = 10 per treatment). In the laboratory experiment, mean diameter growth rates in methylene blue, neutral red, and sea water injected anemone were 0.10, 0.04, and 0.10 mm/day, respectively. In the field experiment, growth rates in methylene blue, neutral red, and sea water injected anemones were 0.0036, 0.0036, and 0.0042 mm/day, respectively, at 40 days. At 156 days, well beyond when methylene blue was visible, diameter growth rates for neutral red and sea water injected anemones were 0.020 and 0.0017 mm/day, respectively. Growth rates were one to two orders of magnitude larger in the laboratory experiment, likely due to the abundant food available in the laboratory setting.

One individual injected with neutral red and one control anemone were lost during the laboratory experiment, but were recovered in the drain trap. They had not died, but were damaged so were excluded from the experiment. Fluorescein at high concentrations (10%) was lethal to *M*. *farcimen*; all anemones died within a week. Anemones marked with 0.25% fluorescein were visibly marked for up to three days. Behavior was qualitatively similar to control anemones in 10% neutral red, 10% methylene blue, and 0.25% fluorescein (i.e., normal movement, feeding, and reaction times to physical stimuli). There could have been depressed growth within the first two weeks due to the injection process, but without a no-injection group this effect is not detectable.

**Fig 2:**
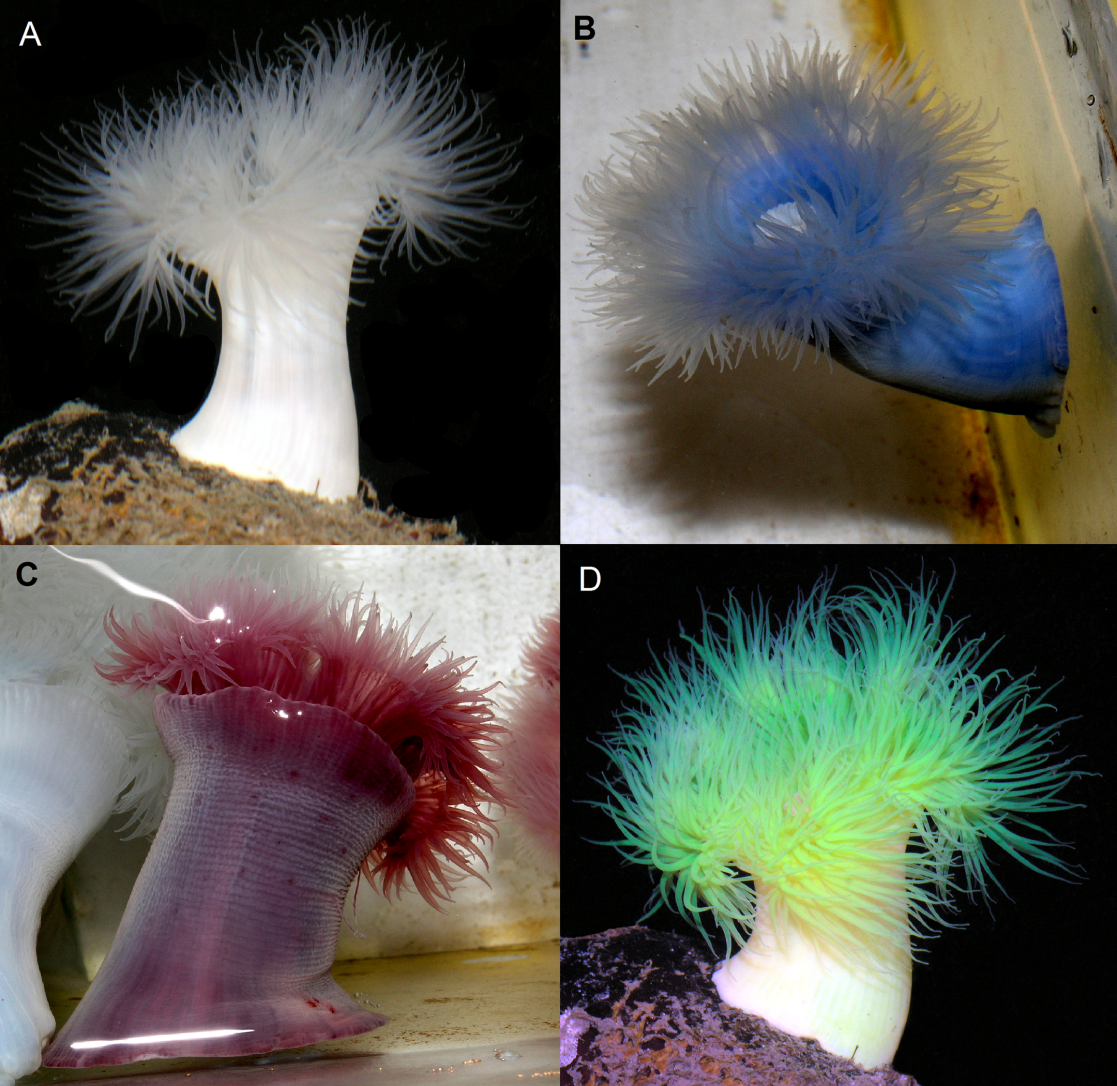
***Metridium farcimen* (A) before injection and injected with 1.0 mL of (B) 10% methylene blue, (C) 10% neutral red, and (D) 0.25% fluorescein.** Endodermal tissue in the column and tentacles as well as cinclides are clearly marked in stained anemones.

**Fig 3:**
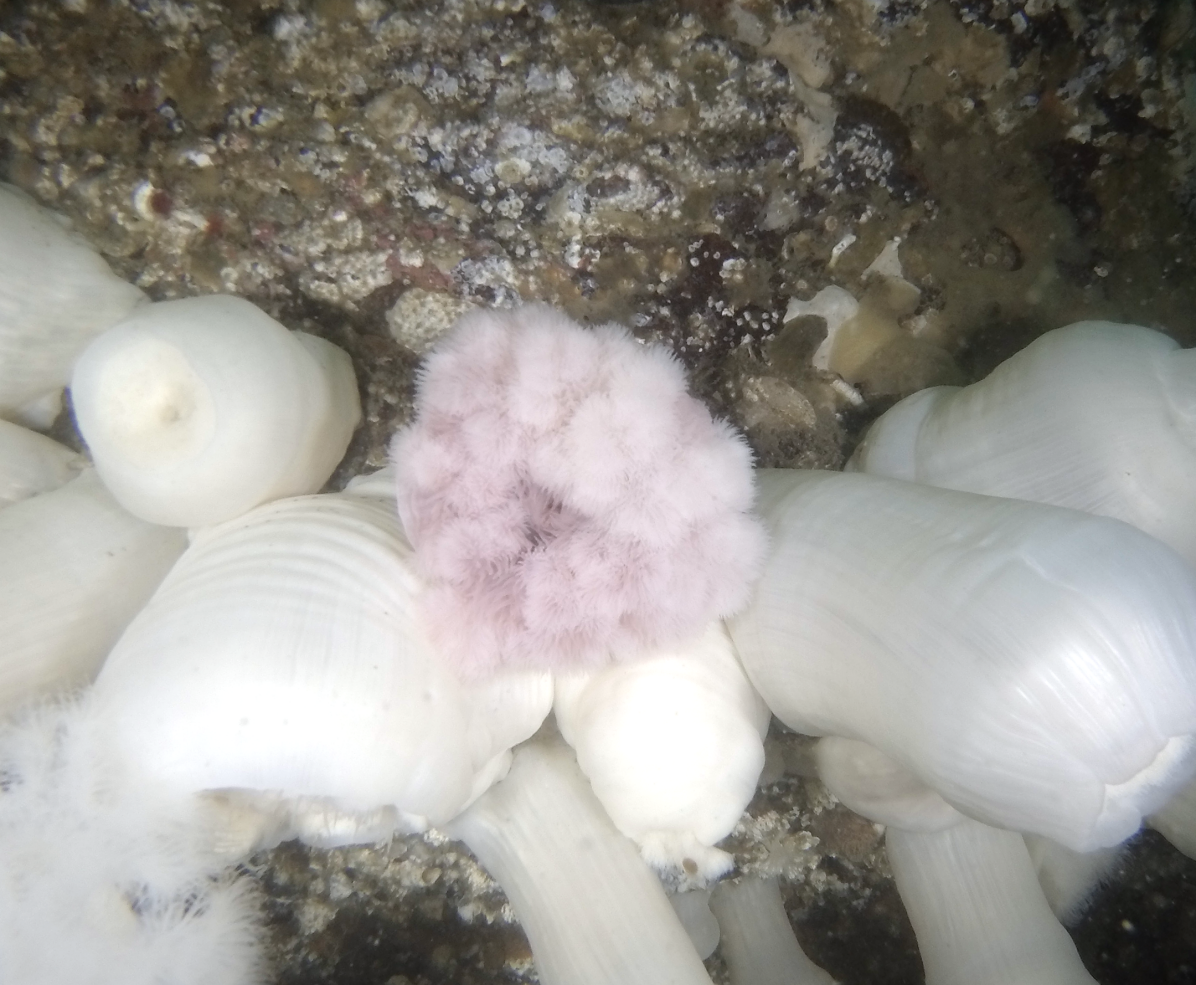
One polyp of *Metridium farcimen* one year after injection with neutral red amongst non-injected individuals.

Marked anemones were clearly stained within seconds, but the mark became brighter and dispersed throughout the animal over the course of an hour as free stain was absorbed from the coelenteron into the tissue. Injecting seawater did not change the color of *M*. *farcimen*. Methylene blue changed the color of the anemones to a brilliant blue (Fig 2B). The majority of the stain was observed bound to endodermal cells and additionally bound to cells surrounding the mouth and cinclides (i.e., blister-like openings to the coelenteron on the column) where anemones ejected coelenteric fluid during the injection process. Marked individuals were recognizable for six weeks, although acontia (i.e., defensive filaments in the coelenteron) kept a blue hue during the full length of the field experiment. With large anemones, acontia coloration was less apparent unless anemones were severely disturbed leading to acontia being extruded out the cinclides, a normal defensive reaction. Injecting anemones with neutral red changed their color a deep red (Fig 2C). Neutral red became noticeably lighter in coloration over time, but marked individuals were recognizable until the conclusion of both experiments. Anemones were revisited two months after the conclusion of the field experiment (seven months post-injection) and marked anemones were still clearly stained. Staining patterns were similar to methylene blue. Neutral red has since been used in subsequent field experiments and neutral red marked anemones have been trackable for over one year (Fig 3). Both 10%, before death, and 0.25% fluorescein injected anemones were a brilliant yellow post-injection with staining patterns similar to the other two stains (Fig 2D).

## Discussion

Both neutral red and methylene blue are effective stains for marking sea anemones in both a field and laboratory setting (Fig 2) with no detectable effect on growth rates in field and laboratory experiments (Fig 1). Neutral red makes an excellent long-term stain as anemones retain this stain for well over six months in this study (Fig 3), and we observed no significant impacts on growth or survival. Due to its shorter retention time (i.e., six weeks), methylene blue can be used for short-term marking of anemones. Combining both neutral red and methylene blue in sea anemones would allow for recognizable patterns for identifying individual anemones (i.e., half blue-half red or purple), although interactive effects of using both dyes simultaneously were not quantified in this study and therefore some caution should be used. Short-term marking of anemones would allow for better tracking of movement and allow for high confidence in identifying individuals in predator-prey experiments with multiple predators or prey. The change in color of the anemones is not a concern for predator-prey studies because predators of anemones (e.g., nudibranchs and sea stars) do not generally rely on visual cues to find prey. Fluorescein is not recommended as a marker as anemones retained marks for minimal time (i.e., three days) at low concentrations and died at high concentrations. This finding was rather unexpected as fluorescein is often used with invertebrates to visualize water flow (e.g., [13–15]). Fluorescein could be used to mark an anemone if marking is only needed for a day (Fig 2).

This method may have utility in other types of sea anemone research in laboratory and field settings, such as labeling tissues for tracing lineages of tissues grafted between anemones (e.g., [16]). Labeling could also be used to track a genet as it asexually reproduces, an extremely common trait in sea anemones (reviewed in [17]), or to track tissues amputated to cause regeneration. This method could be expanded to include corals and other cnidarians, although it is unknown how these stains would interact with the calcified parts of scleractinians and octocorals. It would be particularly interesting to mark the scyphistomae of scyphozoans and examine how tissue is allocated to the budding ephyrae.

While qualitative observations of behavior were recorded during the lab experiment, effects on reproduction, feeding, or other activities were not quantified and it is possible that these dyes could have other unanticipated effects on *M*. *farcimen*. This could be particularly important for future studies that include measures of fitness or other longer-term metrics. Similarly, dye would change an anemone’s albedo, which would be especially important for those in the intertidal zone that are at risk of stress from heat or drying out. Additionally, many anemones associate with algal symbionts and how these stains affect the symbiosis as well as the growth and survival of the symbionts is unknown.

While methylene blue and neutral red make effective markers for sea anemones, the internal impact on other taxa is unknown. Neutral red had no effect on survival of termites when taken up in their water [18]. Submersion of amphipods and gastropods in neutral red, which marked internal structures, did not reduce survival [19, 20]. Both internal and external applications of methylene blue have few to no complications on targeted tissues in humans [21, 22]. External marking with neutral red did have a negative impact on growth and survival in larval amphibians [23, 24] while external application of methylene blue reduces predator avoidance [25]. Exposure to methylene blue and neutral red, separately, affected development, pupation, and survival of female mosquito larvae, but not of males [26].

It is clear that methylene blue, neutral red, and fluorescein can have a diversity of effects on different taxa, ranging from long-term healthy staining to death. Caution should be used whenever applying a marking technique to a new taxon. Impacts of the staining process (e.g., injection, submersion, or taking stain up through food or water) and the stain itself on survival, growth, or any other parameters critical to the experiment must be quantified and addressed before proceeding with other experiments.
